# Structure-Activity Relationship Studies Based on 3D-QSAR CoMFA/CoMSIA for Thieno-Pyrimidine Derivatives as Triple Negative Breast Cancer Inhibitors

**DOI:** 10.3390/molecules27227974

**Published:** 2022-11-17

**Authors:** Jin-Hee Kim, Jin-Hyun Jeong

**Affiliations:** Yonsei Institute of Pharmaceutical Sciences, College of Pharmacy, Yonsei University, Incheon 21983, Republic of Korea

**Keywords:** TNBC, VEGFR3, thieno-pyrimidine derivatives, 3D-QSAR, CoMFA, CoMSIA

## Abstract

Triple-negative breast cancer (TNBC) is defined as a kind of breast cancer that lacks estrogen receptors (ER), progesterone receptors (PR), and human epidermal growth factor receptors (HER2). This cancer accounts for 10–15% of all breast cancers and has the features of high invasiveness and metastatic potential. The treatment regimens are still lacking and need to develop novel inhibitors for therapeutic strategies. Three-dimensional quantitative structure-activity relationship (3D-QSAR) analyses, based on a series of forty-seven thieno-pyrimidine derivatives, were performed to identify the key structural features for the inhibitory biological activities. The established comparative molecular field analysis (CoMFA) presented a leave-one-out cross-validated correlation coefficient q^2^ of 0.818 and a determination coefficient r^2^ of 0.917. In comparative molecular similarity indices analysis (CoMSIA), a q^2^ of 0.801 and an r^2^ of 0.897 were exhibited. The predictive capability of these models was confirmed by using external validation and was further validated by the progressive scrambling stability test. From these results of validation, the models were determined to be statistically reliable and robust. This study could provide valuable information for further optimization and design of novel inhibitors against metastatic breast cancer.

## 1. Introduction

Breast cancer is the most frequently diagnosed cancer that occurs from the epithelium of the ducts or lobules in the breast glandular tissue [1,2]. In 2020, 2.3 million women were diagnosed with breast cancer, and 685,000 of them leading to deaths around the world [3]. There are four subtypes of breast cancer based on the expression of the estrogen receptor (ER), progesterone receptor (PR), and human epidermal growth factor receptor 2 (HER2) [4,5,6,7,8,9]. Triple-negative breast cancer (TNBC), accounting for 10–15% of all breast cancers, is regarded as a heterogeneous and aggressive type of breast cancer with negative expression of ERs, PRs, and HER2s. Compared to receptor-positive breast cancers, TNBC is associated with poorer prognosis and higher rates of recurrence [10].

The vascular endothelial growth factor (VEGF) family participates in the formation of new blood and lymphatic vessels in tumor-related tissue, which leads to the spread of tumor cells [11,12]. VEGF receptor-3 (VEGFR3), as the primary factor in tumor lymphatic angiogenesis, is overexpressed in human breast cancer. The tumor-associated lymphangiogenesis and lymphatic metastasis for breast cancer can be suppressed by the inhibition of VEGFR3 signaling [13,14]. It has been reported that anti-VEGFR3 therapy combined with taxanes targeting lymph nodes has a significant effect on TNBC patients [15].

In the studies by Li et al., N-(4-chloro-3-(trifluoromethyl)phenyl)-4-(6-(4-(4-methylpiperazin-1-yl)phenyl)thieno [2,3-d]pyrimidin-4-yl)piperazine-1-carboxamide **42,** as the novel small molecule VEGFR3 inhibitor, possessed a suitable potency on lymphatic cells and TNBC cells. The most active compound **42** also exhibited higher selectivity (>100) for VEGFR3 compared to other subfamily members such as VEGFR1 and VEGFR2. Based on the interaction analysis of the binding mode between the most active compound **42** and VEGFR3, the Asn934, Arg940, and Arg984 amino acid residues of VEGFR3 were explained to play a key role in the selectivity of the most active compound **42**. The urea NH of the most active compound **42** showed the hydrogen bonding interaction with the carbonyl oxygen of Leu851. The urea oxygen of the most active compound **42** interacted with the amide NH of Asn934 through two hydrogen bonds. The 4-chloro-3-(trifluoromethyl)phenyl group of the most active compound **42** displayed a hydrophobic interaction with Phe929, Ala983, and Leu1044. The benzene ring of the N-methyl-4-(*p*-phenyl)piperazine group of the most active compound **42** formed a hydrophobic interaction with Arg940 by the *π*–cation interaction. The urea group between the rings A and B, the benzene ring E, and the N-methyl-4-(p-phenyl)piperazine group of the most active compound **42** were necessary to increase the biological activity [16].

**Figure 1:**
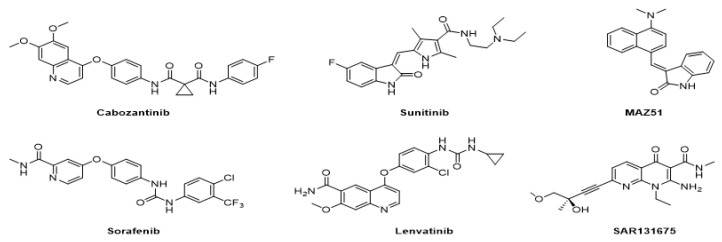
Inhibitors targeting VEGFRs.

The lymphangiogenic inhibitors targeting the VEGFRs are displayed in Figure 1. Cabozantinib and Sorafenib are FDA-approved inhibitors targeting VEGFRs against renal cell carcinoma [17,18,19,20]. Sunitinib is FDA-approved targeting VEGFRs for renal cell carcinoma and presents the reduction of tumor lymphatic and blood vessels against breast cancer in preclinical status [21]. Targeting VEGFR2/3, Lenvatinib is FDA-approved for thyroid cancer and presents the suppression of lymph node metastasis against breast cancer in a preclinical study [22]. In the case of targeting VEGFR3, there are MAZ51 against melanoma and SAR131675 against diabetic nephropathy in preclinical status [23,24].

For the analysis of the structural compound patterns governing the biological activity, computer-aided research is known as a relatively fast and economical technique compared to the time-consuming and costly method of in vitro activity determination. Among structure-activity relationship (SAR) analytical studies, three-dimensional quantitative structure-activity relationship (3D-QSAR) analysis is one of the most useful computational approaches for developing potent and effective inhibitors [25,26]. The 3D-QSAR, using the ligand-based alignment of compounds, is widely used for the identification of effective structural features associated with biological activities. In the 3D-QSAR methods, comparative molecular field analysis (CoMFA) and comparative molecular similarity indices analysis (CoMSIA) by using known biological activities can provide, not only, helpful guidance but also a prediction of the bioactivity for the design of novel potent inhibitors [27,28].

To date, no VEGFR3 selective small molecule inhibitor has been approved by the FDA. It is necessary to develop a highly selective VEGFR3 inhibitor with an anti-VEGFR3 therapeutic effect on breast cancer patients. In the present study, the 3D-QSAR was performed to analyze the correlation between the structural features and the inhibitory activities of the thieno-pyrimidine derivatives as VEGFR3 inhibitors against TNBC [16]. The obtained information from 3D-QSAR analysis could be helpful for the construction of novel potent inhibitors against breast cancer.

## 2. Results and Discussion

### 2.1. 3D-QSAR

The models were generated with all possible combinations of the steric and electrostatic fields for CoMFA and with all possible combinations of steric, electrostatic, hydrophobic, hydrogen bond donor, and hydrogen bond acceptor fields for the CoMSIA. To build the models, the cross-validated partial least square (PLS) method, which provided a leave-one-out cross-validated correlation coefficient q^2^ and determination coefficient r^2^, was used [29]. Based on the basic requirement of the criteria proposed by Golbraikh and Tropsha, the model satisfying q^2^ > 0.5 and r^2^ > 0.6 is considered to be acceptable and predictive [30,31]. Among all constructed models, some models could not meet the criteria of q^2^ > 0.5 and r^2^ > 0.6, which indicates an unacceptable 3D-QSAR model.

As shown in Table 1, CoMFA_SE and CoMSIA_SEHDA models satisfied the q^2^ > 0.5 and r^2^ > 0.6 criteria. The CoMFA model presented a q^2^ of 0.818, r^2^ of 0.917, standard error of estimate (SEE) of 8.142, fisher test value (F) of 114.235, predictive correlation coefficient (r^2^_pred_) of 0.794, and an optimum number of components (ONC) of 3. The contributions of steric and electrostatic fields were 67.7% and 32.3%, respectively. For the CoMSIA model, the statistical results exhibited a q^2^ of 0.801, r^2^ of 0.897, SEE of 9.057, F value of 90.340, r^2^_pred_ of 0.762, and an ONC of 3. The field contributions of steric, electrostatic, hydrophobic, hydrogen bond donor, and hydrogen bond acceptor were 29.5, 29.8, 29.8, 6.5, and 4.4%, respectively. To assess the predictive capability for the generated CoMFA and CoMSIA models, r^2^_pred_ was calculated with the test set compounds. The detailed results for all possible combination models are presented in Table S1 (Supplementary Materials).

#### 2.1.1. CoMFA Studies

##### Scatter Plots

**Figure 2:**
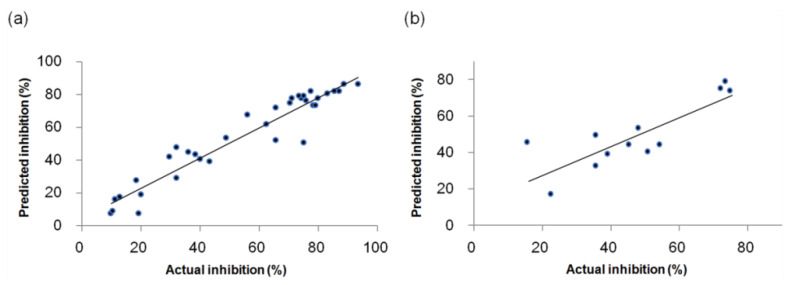
Scatter plots of the actual versus predicted inhibitory activities for the CoMFA model; (**a**) The training set; (**b**) The test set.

The scatter plots between the actual and predicted inhibitory activities of the training and test sets for the CoMFA model are shown in Figure 2. The data distribution of the training and test set compounds are fit well to the line Y = X, which represents that the constructed CoMFA model shows the predictive capability.

##### Progressive Scrambling Stability Test

To validate the robustness of the CoMFA model, the progressive scrambling stability test was performed and the obtained results are shown in Table 2 [32]. The inhibitory activities as the dependent variable were randomly shuffled and a new QSAR model was built. In the stable model, the slope (dq^2^′/dr^2^yy′) would be less than 1.20. The best model was found to be the 3-components model with the highest q^2^ value of 0.691, the lowest cSDEP (calculated cross-validated standard error of prediction) of 15.640, and the slope value of 1.102. The 3-components, as the ideal number of ONC, was identified, which was consistent with having an ONC of 3 in the CoMFA model. It indicates that the developed CoMFA model is robust and statistically reliable.

##### Contour Map Analysis

**Figure 3:**
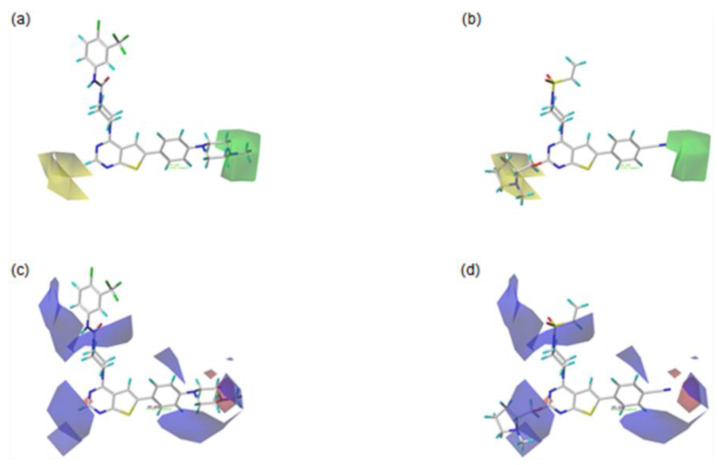
Contour maps of CoMFA analysis with the most active compound **42** (**left**) and the least compound **20** (**right**). In each field, favored and disfavored areas are fixed with 80% and 20% contribution levels, respectively; (**a**,**b**) Steric field: favored regions are in green contours and disfavored regions are in yellow contours; (**c**,**d**) Electrostatic field: favored regions are in blue contours and disfavored regions are in red contours.

For the visualization and the explanation of each field effect, the CoMFA contour maps created by the StDev*Coeff mapping option are superimposed with the most active compound **42**, and the least active compound **20** in Figure 3. The contributions for the favorable and unfavorable regions in each field are displayed by 80% and 20%, respectively.

In the steric contour maps, as shown in Figure 3a for the most active compound **42** and **3b** for the least active compound **20**, the green contour block means that the presence of the bulky steric groups in this area would be favorable for the biological activity of a compound, while the yellow block represents that the bulky groups would be unfavorable. The green contour block covering the F ring of the most active compound **42** in Figure 3a indicates that the bulky group in this area would be favorable to increase the bioactivity of a compound. For example, compound **5** with the bulky methyl morpholine group on the green region is more biologically active than compound **3** with the methoxy group. In addition, this could explain why compound **6** with the bulkier methyl piperazine group on the green area is more active than compound **5** with the morpholine group. Compared to compound **4**, with no substituent on the yellow block, compounds **20** and **21**, with the large substituents on this block, are less active. Moreover, compound **22** with the bulky substituted group on the yellow area shows lower biological activity than compound **7**, with no substituted group in this region. This could explain why the compounds **23**, **24**, **25**, **26**, **45**, **46**, and **47** with the large substituent on the yellow region show the reduced activities.

As shown in Figure 3c for the most active compound **42** and Figure 3d for the least active compound **20**, the blue contours represent that the electropositive groups would be favorable to increase the activity of a compound, while the red contours indicate that the electronegative groups would be unfavorable to the activity of a compound. Compared to compound **6**, with no substituent group on the blue contour, compound **25**, with the electronegative groups on this block, is less active. This could explain why compounds **20**, **21**, **22**, **23**, **24**, **25**, **26**, **45**, **46**, and **47** present low activities due to the presence of the electronegative substituents on the blue contour region. The compounds **4**, **5**, and **6**, with electronegative groups on the red contour area, show the higher bioactivities than compound **1**, without substituents at the corresponding region.

#### 2.1.2. CoMSIA Studies

##### Scatter Plots

**Figure 4:**
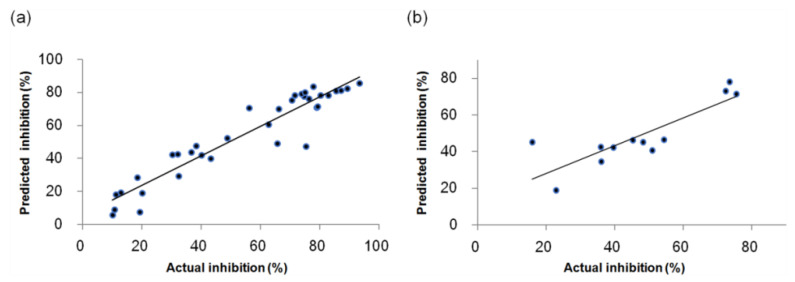
Scatter plots of the actual versus predicted inhibitory activities for the CoMSIA model; (**a**) The training set; (**b**) The test set.

In Figure 4, the scatter plots between the actual and predicted inhibitory activities of the training, and test sets for the CoMSIA model, are shown. The data correlation of the training and test sets shows a good fit along the fitted line, which indicates that the developed CoMSIA model has a predictable ability.

##### Progressive Scrambling Stability Test

The progressive scrambling stability test was carried out for the additional validation of the CoMSIA model. As shown in Table 3, the obtained results of this validation demonstrate that the 3-components model was found to be the best model with the highest q^2^ value of 0.674, the lowest cSDEP (calculated cross-validated standard error of prediction) of 16.106, and the slope value of 1.035. This ONC of 3 was identified and consistent with the ONC of 3 in the generated CoMSIA model, which means that the CoMSIA model is statistically stable and robust.

##### Contour Map Analysis

The CoMSIA steric and electrostatic contour maps are similar to those of the CoMFA discussed above. Therefore, the following discussion is focused on the contour maps of the hydrophobic, hydrogen bond donor, and hydrogen bond acceptor fields.

**Figure 5:**
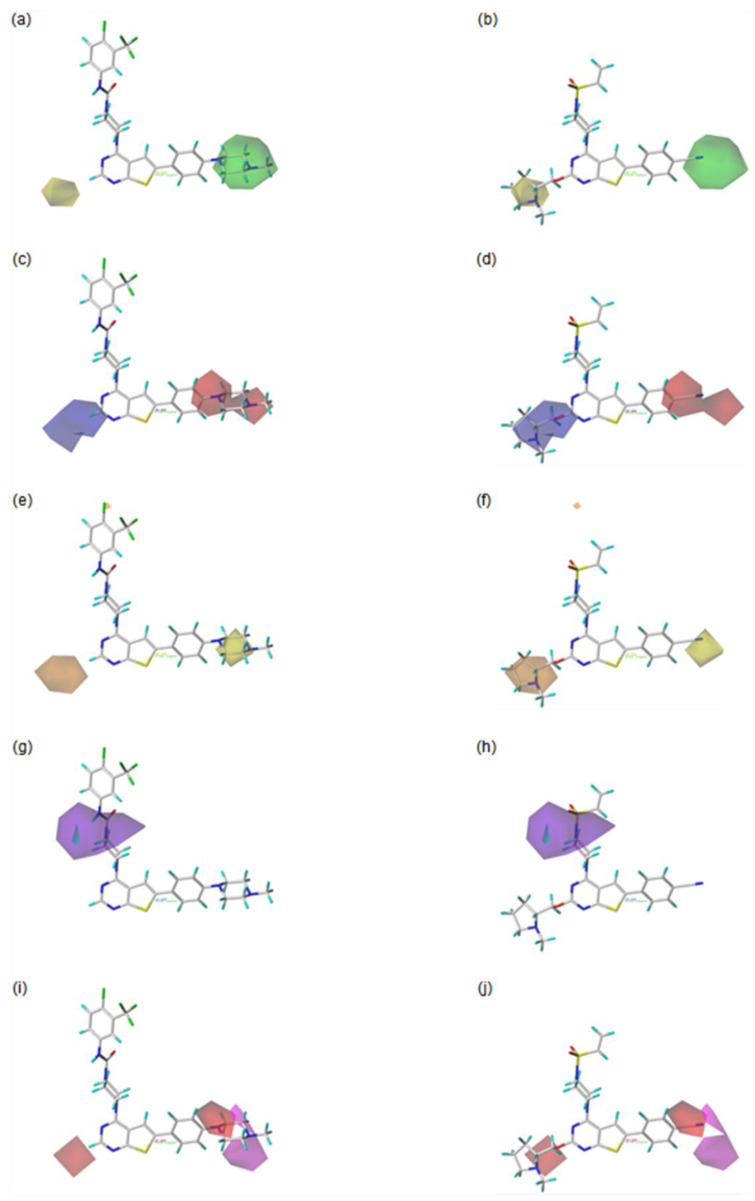
Contour maps of CoMSIA with the most active compound **42** (**left**) and the least active compound **20** (**right**). In each field, favored and disfavored areas are fixed with 80% and 20% contribution levels, respectively; (**a**,**b**) Steric field: favored regions are in green contours and disfavored regions are in yellow contours; (**c**,**d**) Electrostatic field: favored regions are in blue contours and disfavored regions are in red contours; (**e**,**f**) Hydrophobic field: favored regions are in orange contours and disfavored regions are in yellow contours; (**g**,**h**) Hydrogen bond donor field: favored regions are in cyan contours and disfavored regions are in purple contours; (**i**,**j**) Hydrogen bond acceptor field: favored regions are in magenta contours and disfavored regions are in red contours.

The contour maps for the hydrophobic field are shown in Figure 5e for the most active compound **42** and Figure 5f for the least active compound **20**. The orange contour blocks indicate that the introduction of the hydrophobic substituent would be favorable for enhancing the biological activity of a compound, while the yellow contours mean that the hydrophobic groups would be unfavorable in this area. For example, compounds **20** and **21**, with the hydrophilic groups including the N or O atom around the orange region, are less active than compound **4**, with hydrogen at the corresponding position. This could explain why compounds **22**, **23**, **24**, **25**, **26**, **45**, **46**, and **47**, with the hydrophilic groups around the orange area, show low biological activities. On the other hand, compound **6** has higher bioactivity than compound **1**. This might be because of the hydrophilic piperazine group of compound **6** on the yellow region, compared to compound **1** with an H atom at the corresponding location.

Figure 5g for the most active compound **42** and Figure 5h for the least active compound **20** display the hydrogen bond donor contour maps. The cyan contours indicate that the hydrogen bond donor group would be favored for improving the bioactivity of a compound, while the purple contours represent that the hydrogen bond donor group would be disfavored for bioactivity in this region. In the most active compound **42**, the presence of the NH group as a hydrogen bond donor group toward the cyan contour block might be one of the factors that lead to high bioactivity.

In the hydrogen bond acceptor contour map, as shown in Figure 5i, for the most active compound **42** and Figure 5j, for the least active compound **20**, the magenta contours represent the area where the hydrogen bond acceptor substituents are favorable for the compound’s bioactivity, while the red contours indicate the region where the hydrogen bond acceptor groups are unfavorable. For example, compound **42** with the N atom, next to the methyl group of the piperazine substituent at the F ring, as the hydrogen bond acceptor around the magenta contour area, has high bioactivity. On the other hand, compounds **20** and **21**, with the N or O atom as the hydrogen bond acceptor around the disfavored red contour region, show lower biological activity than compound **4** with the H atom at the corresponding area. This could explain why the compounds **22**, **23**, **24**, **25**, **26**, **45**, **46**, and **47** with the N or O atom as the hydrogen bond acceptor at the red contour region have low bioactivities.

#### 2.1.3. Design of the Novel Potent Derivatives

Based on the information provided by the developed 3D-QSAR models, CoMFA_SE and CoMSIA_SEHDA, we have designed a series of novel potent thieno-pyrimidine derivatives. The chemical structures and predicted biological activities of the newly designed inhibitors are presented in Table 4. The newly designed compounds **N1**–**N14** were aligned to the database using compound **42** as the template and their inhibitory activities were predicted. They showed higher bioactivities than that of the most active compound **42** in the series. The introduction of bulky substituents on the piperazine ring F and the modification of the bulky substituents, including the carbonyl group or the sulfonyl group at one of the N atoms of the piperazine ring F, could improve the inhibitory activities of the novel designed compounds **N1**–**N14** more than those of the most active compound **42** in the series.

To support drug development, we have also predicted the ADMET (absorption, distribution, metabolism, excretion, and toxicity) parameters, pharmacokinetic properties, drug-likeness nature, and synthetic accessibility of the novel designed compounds **N1**–**N14** using the online servers SwissADME [33] and pkCSM [34]. The detailed properties of the most active compound **42** and the newly designed inhibitors **N1**–**N14** are shown in Table 5. The rule-based Moriguchi log P (MLogP) value was calculated for the lipophilicity and the log S by estimated solubility (ESOL) was calculated for the solubility. The gastrointestinal (GI) absorption and the blood-brain barrier (BBB) permeant were also calculated. Toxicity was predicted by Ames positive test to assess the mutagenic potential of compounds to act as a carcinogen. Drug-likeness qualitatively evaluates the potential of a compound to be an oral drug with respect to bioavailability. Two or more Lipinski’s rule-of-five (Ro5) violations predict a compound as a non-orally available drug. Lipinski’s Ro5 violation defines four classes of molecules with the probabilities of 11%, 17%, 56%, and 85%. The novel designed inhibitors **N1**–**N14** presented the prediction results of the acceptable ADMET properties. To represent ease of synthesis, the synthetic accessibility as a score from 1 (very easy to make) to 10 (very difficult to make) is described. The SMILES codes for the most active compound **42** and the novel designed derivatives N1-N14 are shown in Table S2 (Supplementary Materials).

## 3. Materials and Methods

### 3.1. Dataset

A series of thieno-pyrimidine derivatives as selective VEGFR3 inhibitors identified by Li et al. were taken for the 3D-QSAR study [16]. The structures and their inhibitory activities are listed in Table 6. To develop the CoMFA and CoMSIA models, forty-seven thieno-pyrimidine analogs were used and divided into thirty-five compounds (74.5%) as the training set and twelve compounds (25.5%) as the test set, based on the structural and bioactive diversity by the DIVERSITY function of SYBYL-X 2.1.1 (Tripos, Inc., St. Louis, MO, USA). The compound structures in the test set sufficiently represented the diversity of the whole dataset. The training set was used for the construction of the model and the test set was used for the validation of the predicted power of the developed model.

### 3.2. Alignment

**Figure 6:**
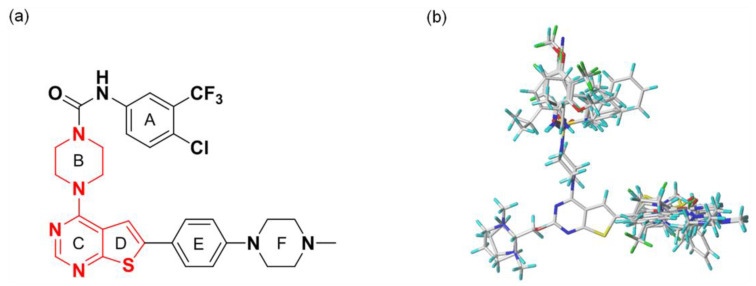
(**a**) The structure of the most active compound 42 as the template including the maximum common substructure in red; (**b**) The alignment of the training and test sets used in the 3D-QSAR model.

All compounds in the training and test sets were drawn in ChemDraw. All compounds were superimposed and aligned on the maximum common substructure of thieno-pyrimidine derivatives by using DISTIL RIGID alignment function in SYBYL-X 2.1.1 [35]. Based on the hypothesis that the common alignment core contributes equally to the bioactivities of the compounds, the conformational angles for maximum common substructures of the most active compound **42** as the template for the alignment were copied and applied to the remaining compounds in the whole dataset. Figure 6 shows the most active compound **42** as the template, and the structural alignment of the training and test sets for the 3D-QSAR study.

### 3.3. CoMFA Studies

The CoMFA was performed by the Lennard–Jones potential for the steric field and Coulombic potential for the electrostatic field in SYBYL-X 2.1.1. The aligned compounds were placed in a 3D cubic lattice of 2.0 Å grid spacing in the x, y, and z directions and these potentials were calculated for each compound. The sp^3^ carbon probe with the van der Waals radius of 1.52 Å and the point charge of +1.0 was used at each lattice point of the grid box for the calculation of the steric and electrostatic fields. To avoid overpower by large steric and electrostatic energy values, the default energy cutoff value was set to 30 kcal/mol. A default value of 0.3 was set as the attenuation coefficient. The regression analysis was performed using the cross-validated partial least square (PLS) method. To develop the final model by performing a PLS analysis, the first run was conducted with the cross-validation to find ONC, and then the final run yielded the non-cross-validated r^2^ value.

### 3.4. CoMSIA Studies

In this study, the CoMSIA model was developed with the steric, electrostatic, hydrophobic, hydrogen bond donor, and hydrogen bond acceptor fields in SYBYL-X 2.1.1. As the five descriptors, the default settings of the probe atom with 1.0 Å radius, +1.0 charge, +1.0 hydrophobicity, +1.0 hydrogen bond donor, and +1.0 hydrogen bond acceptor properties were used in the same grid box for the CoMFA calculation. The default attenuation coefficient was set to 0.3. In CoMSIA, the Gaussian function was used for the distance dependence between the probe atom and the molecule atoms to measure the relative attenuation of the field position for each atom in the lattice. The similarity indices were calculated at all grid points through the different shape of the Gaussian function. The use of the Gaussian function resulted in smoother sampling of the fields around the molecules compared to CoMFA.

### 3.5. Partial Least Squares Analysis

The partial least squares analysis (PLS) regression method was performed to analyze the linear correlation of CoMFA and CoMSIA as the independent variables to the biological activities as the dependent variables. The sample-distance partial least square (SAMPLS) method in SYBYL-X2.1.1 was used to speed up the cross-validation analysis. Cross-validation analysis was carried out by using the leave-one-out (LOO) method, which removes one compound from the training set and predicts its bioactivity from the constructed model using the rest compounds. Until all compounds have been removed once, this process is repeated to obtain the cross-validated correlation coefficient q^2^, the optimum number of components (ONC). To speed up the analysis and reduce noise, a minimum column filtering value was set to 2.0 Kcal/mol. Using the determined ONC, the final PLS model was constructed without cross-validation. The predictive ability of the developed 3D-QSAR models was confirmed by external validation using the test set compounds, which were not included in the training set. For the test set compounds, the optimization, alignment, and all other steps were the same as those of the training set compounds, and then their biological activities were predicted using the final model developed by the training set compounds. The predictive correlation coefficient r^2^pred value is obtained by this formula:

r^2^_pred_ = (SD − PRESS)/(SD)

In the formula, SD represents the sum of the squared deviations between the inhibitory activities in the test set and the mean inhibitory activity in the training set, and PRESS represents the sum of the squared deviations between the actual and predicted inhibitory activities of the test set compounds.

### 3.6. Progressive Scrambling Stability Test

To further validate the robustness and stability of the developed model, the progressive scrambling stability test was carried out by the Scrambling Stability Test function in SYBYL-X 2.1.1 [32]. The sensitivity of the model to small systematic perturbations was determined by this test. The inhibitory activities of the training set compounds were randomly scrambled many times and the q^2^ values were generated using LOO cross-validation after every iteration. The unstable 3D-QSAR model is characterized by a greater slope (dq^2^′/dr^2^yy′) value than 1.2.

## 4. Conclusions

**Figure 7:**
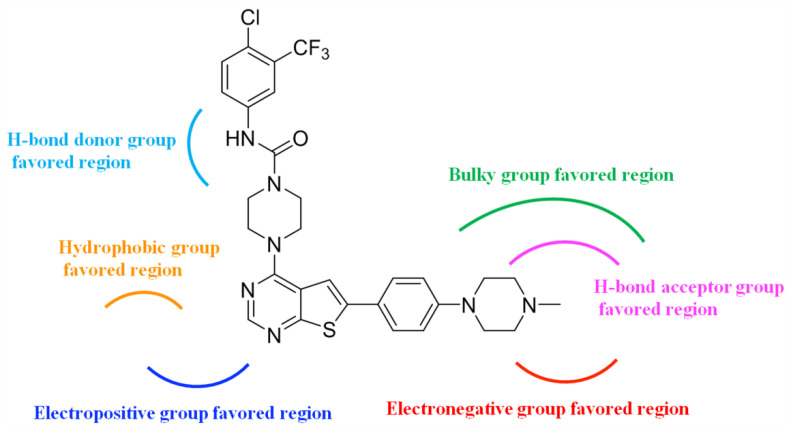
The novel design strategy information obtained from the 3D-QSAR study.

In the present study, 3D-QSAR analyses were performed to identify the key structural features for improving the biological activities of the thieno-pyrimidine derivatives as breast cancer inhibitors. The CoMFA (q^2^ = 0.818, r^2^ = 0.917) and CoMSIA (q^2^ = 0.801, r^2^ = 0.897) models, satisfying the criteria provided by Golbraikh and Tropsha, were selected. Statistical reliability and robustness of the selected CoMFA and CoMSIA models were confirmed by external validation with the test set compounds which did not exist in the training set. The established models were further validated by the progressive scrambling stability test and presented to be statistically stable and robust. The contour maps of CoMFA and CoMSIA provided key information to understand the SAR and to identify the structural requirement for enhancing the inhibitory bioactivity of compounds. Based on the information derived from the contour maps, the summarized design strategy of the principal SAR for the design of the new compounds, with potential therapeutic applications, is shown in Figure 7. These obtained results might be helpful in the design of novel inhibitors with improved inhibitory biological activity against metastatic breast cancer.

## Figures and Tables

**Table 1:** Statistical results of the selected CoMFA and CoMSIA models.

Model	q^2^	r^2^	SEE	F	r^2^_pred_	ONC	Field Contribution (%)				
							S	E	H	D	A
CoMFA_SE	0.818	0.917	8.142	114.235	0.794	3	67.7	32.3	-	-	-
CoMSIA_SEHDA	0.801	0.897	9.057	90.340	0.762	3	29.5	29.8	29.8	6.5	4.4

Leave-one-out cross-validated correlation coefficient (q^2^), determination coefficient (r^2^), standard error of estimate (SEE), Fisher test value (F), predictive correlation coefficient (r^2^_pred_), optimum number of components (ONC), Steric (S), Electrostatic (E), Hydrophobic (H), Hydrogen bond donor (D), Hydrogen bond acceptor (A).

**Table 2:** Summary of progressive scrambling stability test for the CoMFA model.

No. of Components	q^2^	cSDEP	dq^2^′/dr^2^yy′
1	0.625	16.763	0.881
2	0.678	15.710	1.058
3	0.691	15.640	1.102
4	0.657	16.722	1.260
5	0.652	17.073	1.266

The predictivity of the model (q^2^ = 1 − (cSDEP)^2^), the calculated cross-validated standard error (cSDEP), and the slope of q^2^ with respect to the correlation of the perturbed dependent variables against the unperturbed data (dq^2^′/dr^2^yy′).

**Table 3:** Summary of progressive scrambling stability test for the CoMSIA model.

No. of Components	q^2^	cSDEP	dq^2^′/dr^2^yy′
1	0.428	20.708	0.709
2	0.644	16.580	0.819
3	0.674	16.106	1.035
4	0.635	17.242	1.162
5	0.593	18.441	1.165

The predictivity of the model (q^2^ = 1 − (cSDEP)^2^), the calculated cross-validated standard error (cSDEP), the slope of q^2^ with respect to the correlation of the perturbed dependent variables against the unperturbed data (dq^2^′/dr^2^yy′).

**Table 4:** The structures and the predicted inhibitory activities for the most active compound **42** and the newly designed inhibitors **N1**–**N14**.

No	Structure	Predicted Inhibitory Activity (%)
42	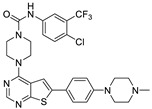	85.61
N1	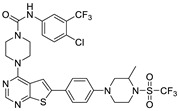	87.11
N2	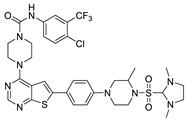	88.01
N3	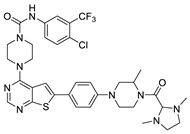	89.16
N4	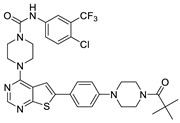	88.19
N5	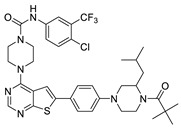	91.18
N6	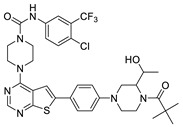	90.94
N7	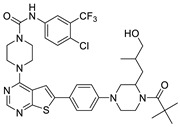	91.23
N8	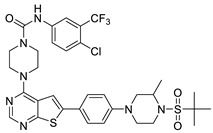	90.10
N9	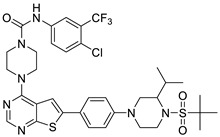	90.77
N10	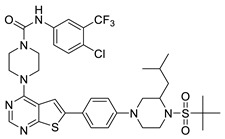	90.87
N11	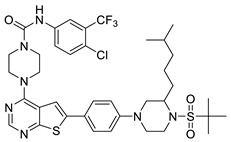	90.45
N12	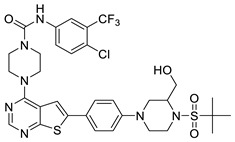	89.13
N13	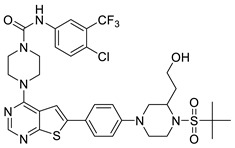	90.44
N14	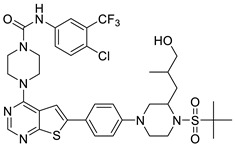	90.55

**Table 5:** The prediction results of ADMET parameters, pharmacokinetic properties, and drug-likeness of the most active compound **42** and the novel designed inhibitors **N1**–**N14**.

No	Lipophilicity Log P_o/w_ (MLOGP)	Water Solubility Log S (ESOL)	Pharmacokinetics			Drug-Likeness Lipinski Rule	Synthetic Accessibility
			GI Absorption	BBB Permeant	Toxicity (AMES) Categorical (Yes/No)		
42	4.05	−7.21	High	No	No	Yes; 1 violation	4.25
N1	3.63	−8.40	Low	No	No	Yes; 1 violation	5.01
N2	2.97	−7.88	Low	No	No	No; 2 violation	5.70
N3	3.56	−7.73	High	No	No	No; 2 violation	5.45
N4	4.34	−8.01	Low	No	No	No; 2 violation	4.66
N5	5.04	−9.31	Low	No	Yes	No; 2 violation	5.57
N6	3.92	−8.08	Low	No	No	Yes; 1 violation	5.63
N7	4.27	−8.57	Low	No	No	No; 2 violation	5.86
N8	3.90	−8.19	Low	No	No	Yes; 1 violation	5.37
N9	4.26	−8.89	Low	No	No	No; 2 violation	5.60
N10	4.43	−9.13	Low	No	No	No; 2 violation	5.73
N11	4.78	−9.73	Low	No	No	No; 2 violation	5.99
N12	3.14	−7.55	Low	No	No	No; 2 violation	5.43
N13	3.32	−7.79	Low	No	No	No; 2 violation	5.52
N14	3.67	−8.39	Low	No	No	No; 2 violation	6.03

**Table 6:** Chemical structures and inhibitory activity values of thieno-pyrimidine derivatives.

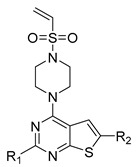		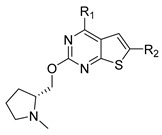			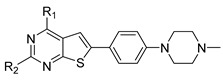		
**1–21**		**22–26, 45–46**			**27–44, 47**		
**No**	**R_1_**	**R_2_**	**Actual** **Inhibitory Activity** **(1** **µM, %)**	**C** **oMFA** **_SE**	**C** **oMSIA** **_SEHDA**	**C** **oMFA Residual**	**C** **o** **MSIA** **Residual**
1 *	H	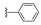	54.23	44.45	46.49	9.78	7.74
2	H	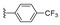	32.10	47.91	42.69	−15.81	−10.59
3	H	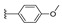	48.68	53.15	52.22	−4.47	−3.54
4	H	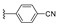	75.36	50.87	47.48	24.50	27.88
5	H	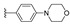	56.16	67.32	70.79	−11.16	−14.63
6	H	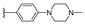	78.87	72.88	71.03	5.99	7.84
7	H	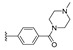	62.70	61.54	60.58	1.16	2.12
8	H	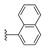	40.11	39.95	42.03	0.16	−1.92
9	H	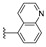	43.07	38.20	40.04	4.87	3.03
10 *	H	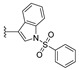	35.91	49.68	42.80	−13.77	−6.89
11	H	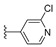	38.28	42.53	47.64	−4.25	−9.36
12	H	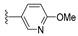	65.71	51.82	49.23	13.89	16.48
13	H	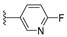	36.60	45.07	43.66	−8.47	−7.06
14 *	H	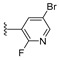	45.18	43.36	46.31	1.82	−1.13
15 *	H	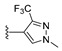	51.02	39.52	40.81	11.50	10.21
16 *	H	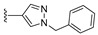	48.28	52.31	45.34	−4.03	2.94
17 *	H	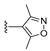	39.59	38.53	42.56	1.06	−2.97
18	H	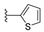	30.18	41.61	42.33	−11.43	−12.15
19 *	H	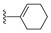	15.99	45.46	45.31	−29.47	−29.32
20	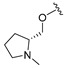	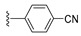	10.01	6.72	5.82	3.29	4.19
21	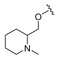	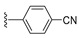	10.61	8.37	8.87	2.24	1.74
22 *	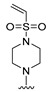	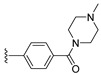	22.80	17.10	18.83	5.70	3.97
23	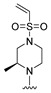	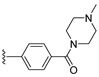	11.20	16.11	18.09	−4.91	−6.89
24	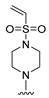	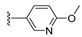	19.22	7.74	7.54	11.48	11.68
25	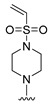	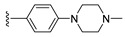	32.34	28.40	29.33	3.94	3.01
26	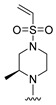	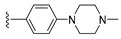	18.33	27.32	28.64	−8.99	−10.31
27	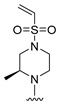	H	66.15	71.53	70.31	−5.38	−4.16
28	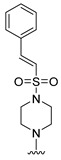	H	82.97	80.21	78.64	2.76	4.33
29	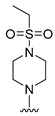	H	79.21	73.30	71.67	5.91	7.54
30 *	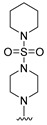	H	72.39	74.94	73.26	−2.55	−0.87
31 *	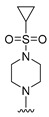	H	75.36	73.07	71.73	2.29	3.63
32	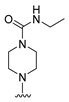	H	76.44	76.16	76.19	0.28	0.25
33	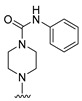	H	80.42	78.12	78.41	2.30	2.01
34 *	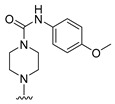	H	73.53	79.37	78.08	−5.84	−4.55
35	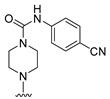	H	89.26	86.34	82.41	2.92	6.86
36	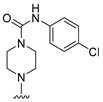	H	85.42	81.22	80.99	4.20	4.43
37	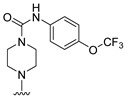	H	87.19	81.94	81.34	5.25	5.85
38	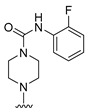	H	74.78	76.84	77.65	−2.05	−2.87
39	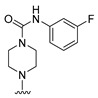	H	71.60	77.42	78.20	−5.82	−6.60
40	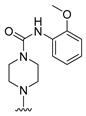	H	70.53	73.91	75.48	−3.38	−4.95
41	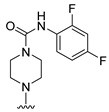	H	73.78	79.07	79.41	−5.29	−5.63
42	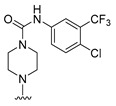	H	93.47	85.84	85.61	7.63	7.86
43	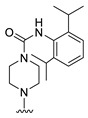	H	75.09	78.32	80.09	−3.23	−4.99
44	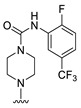	H	77.82	82.12	83.61	−4.30	−5.79
45	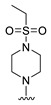	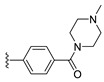	12.80	17.51	19.46	−4.71	−6.66
46	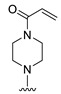	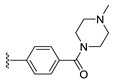	20.08	19.20	19.08	0.88	1.00
47 *	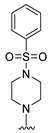	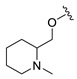	36.09	31.69	34.75	4.40	1.34

* Compounds in the test set.

## Data Availability

Not applicable.

## Supplementary Materials

The following supporting information can be downloaded at: https://www.mdpi.com/article/10.3390/molecules27227974/s1, Table S1: Statistical results of all possible field combinations for the CoMFA and CoMSIA models. Table S2. The SMILES codes for the most active compound **42** and the novel designed inhibitors **N1**–**N14**.

## Sample Availability

Samples of the compounds are not available from the authors.
