# Supplementary material for: Structure-Activity Relationship Studies Based on 3D-QSAR CoMFA/CoMSIA for Thieno-Pyrimidine Derivatives as Triple Negative Breast Cancer Inhibitors
Source: Molecules. 2022 Nov 17;27(22):7974. doi: 10.3390/molecules27227974 (PMC9698756; doi:10.3390/molecules27227974)
Supplement: Supplementary file 1 [file molecules-27-07974-s001.zip › molecules-1995218-supplementary-proofdone.pdf]

Supplementary materials

Structure-Activity Relationship Studies Based on 3D-QSAR CoMFA/CoMSIA for Thieno-pyrimidine Derivatives as Triple Negative Breast Cancer Inhibitors

Jin-Hee Kim and Jin-Hyun Jeong*

Table S1. Statistical results of all possible field combinations for the COMFA and COMSIA models.

							Field Contribution (%)				
**Model**	**q^2**	**r^2**	**SEE**	**F**	**r^2_{pred}**	**ONC**	**S**	**E**	**H**	**D**	**A**
COMFA_S	0.859	0.937	7.215	111.472	0.840	4	100	-	-	-	-
COMFA_E	0.800	0.941	7.079	93.081	0.804	5	-	100	-	-	-
COMFA_SE	0.818	0.917	8.142	114.235	0.794	3	67.7	32.3	-	-	-
COMSIA_S	0.845	0.871	9.826	223.576	0.750	1	100	-	-	-	-
COMSIA_E	0.800	0.961	5.849	116.027	0.970	6	-	100	-	-	-
COMSIA_H	0.820	0.899	9.001	91.600	0.792	3	-	-	100	-	-
COMSIA_D	0.357	0.418	20.894	23.748	0.238	1	-	-	-	100	-
COMSIA_A	0.423	0.569	18.270	21.108	0.573	2	-	-	-	-	100
COMSIA_SE	0.822	0.935	7.313	108.295	0.889	4	42.0	58.0	-	-	-
COMSIA_SH	0.836	0.901	8.917	93.522	0.793	3	49.2	-	50.8	-	-
COMSIA_SD	0.846	0.890	9.522	60.811	0.804	4	91.0	-	-	9.0	-
COMSIA_SA	0.814	0.887	9.513	80.915	0.784	3	83.2	-	-	-	16.8
COMSIA_EH	0.797	0.943	6.841	124.826	0.878	4	-	53.4	46.6	-	-
COMSIA_ED	0.806	0.969	5.525	87.406	1.026	9	-	83.0	-	17.0	-
COMSIA_EA	0.749	0.923	7.995	89.393	0.777	4	-	83.5	-	-	16.5
COMSIA_HD	0.817	0.898	9.177	66.030	0.774	4	-	-	95.0	5.0	-
COMSIA_HA	0.780	0.888	9.464	81.861	0.760	3	-	-	85.9	-	14.1
COMSIA_DA	0.428	0.594	18.007	15.133	0.523	3	-	-	-	49.1	50.9
COMSIA_SEH	0.817	0.907	8.633	100.469	0.794	3	32.8	33.5	33.7	-	-
COMSIA_SED	0.816	0.910	8.604	76.150	0.804	4	44.9	49.2	-	5.9	-
COMSIA_SEA	0.798	0.899	8.999	91.629	0.769	3	44.7	44.8	-	-	10.5
COMSIA_SHD	0.834	0.901	9.043	68.229	0.793	4	46.8	-	48.5	4.6	-
COMSIA_SHA	0.819	0.897	9.080	89.818	0.782	3	45.2	-	46.2	-	8.7
COMSIA_SDA	0.807	0.888	9.638	59.168	0.777	4	79.9	-	-	9.2	10.9
COMSIA_EHD	0.798	0.914	8.443	79.386	0.782	4	-	47.5	47.4	5.1	-
COMSIA_EHA	0.774	0.898	9.012	91.333	0.751	3	-	44.6	47.1	-	8.2
COMSIA_EDA	0.734	0.931	7.693	77.906	0.807	5	-	77.4	-	8.7	13.9
COMSIA_HDA	0.775	0.892	9.459	61.713	0.753	4	-	-	81.8	6.6	11.7
COMSIA_SEHD	0.815	0.903	8.819	95.847	0.785	3	31.1	32.1	31.5	5.3	-
COMSIA_SEHA	0.805	0.902	8.838	95.392	0.775	3	31.0	30.9	31.8	-	6.2
COMSIA_SEDA	0.793	0.895	9.173	87.807	0.749	3	42.0	42.6	-	8.3	7.2
COMSIA_SHDA	0.816	0.892	9.273	85.697	0.778	3	42.5	-	42.2	8.3	7.0
COMSIA_EHDA	0.767	0.894	9.216	86.898	0.735	3	-	43.0	43.2	7.4	6.3
COMSIA_SEHDA	0.801	0.897	9.057	90.340	0.762	3	29.5	29.8	29.8	6.5	4.4

leave-one-out cross-validated correlation coefficient (q^2), determination coefficient (r^2), standard error of estimate (SEE), Fisher test value (F), predictive correlation coefficient (r^2_{pred}), optimum number of components (ONC), Steric (S), Electrostatic (E), Hydrophobic (H), Hydrogen bond donor (D), Hydrogen bond acceptor (A).

Table S2. The SMILES codes for the most active compound **42** and the novel designed inhibitors **N1**-**N14**.

No	SMILES Structure
42	CN(CC1)CCN1C(C=C2)=CC=C2C3=CC4=C(N5CCN(C(NC6=CC=C(Cl)C(C(F)(F)F)=C6)=O)CC5)N=CN=C4S3
N1	O=C(NC1=CC=C(Cl)C(C(F)(F)F)=C1)N(CC2)CCN2C3=C4C(SC(C5=CC=C(N6CCN(S(=O)(C(F)(F)F)=O)C(C)C6)C=C5)=C4)=NC=N3
N2	O=C(NC1=CC=C(Cl)C(C(F)(F)F)=C1)N(CC2)CCN2C3=C4C(SC(C5=CC=C(N6CCN(S(C7N(C)CCN7C)(=O)=O)C(C)C6)C=C5)=C4)=NC=N3
N3	O=C(NC1=CC=C(Cl)C(C(F)(F)F)=C1)N(CC2)CCN2C3=C4C(SC(C5=CC=C(N6CCN(C(C7N(C)CCN7C)=O)C(C)C6)C=C5)=C4)=NC=N3
N4	O=C(NC1=CC=C(Cl)C(C(F)(F)F)=C1)N(CC2)CCN2C3=C4C(SC(C5=CC=C(N6CCN(C(C(C)(C)C)=O)CC6)C=C5)=C4)=NC=N3
N5	O=C(NC1=CC=C(Cl)C(C(F)(F)F)=C1)N(CC2)CCN2C3=C4C(SC(C5=CC=C(N6CCN(C(C(C)(C)C)=O)C(CC(C)C)C6)C=C5)=C4)=NC=N3
N6	O=C(NC1=CC=C(Cl)C(C(F)(F)F)=C1)N(CC2)CCN2C3=C4C(SC(C5=CC=C(N6CCN(C(C(C)(C)C)=O)C(C(C)O)C6)C=C5)=C4)=NC=N3
N7	O=C(NC1=CC=C(Cl)C(C(F)(F)F)=C1)N(CC2)CCN2C3=C4C(SC(C5=CC=C(N6CCN(C(C(C)(C)C)=O)C(CC(C)CO)C6)C=C5)=C4)=NC=N3
N8	O=C(NC1=CC=C(Cl)C(C(F)(F)F)=C1)N(CC2)CCN2C3=C4C(SC(C5=CC=C(N6CCN(S(C(C)(C)C)(=O)=O)C(C)C6)C=C5)=C4)=NC=N3
N9	O=C(NC1=CC=C(Cl)C(C(F)(F)F)=C1)N(CC2)CCN2C3=C4C(SC(C5=CC=C(N6CCN(S(C(C)(C)C)(=O)=O)C(C(C)C)C6)C=C5)=C4)=NC=N3
N10	O=C(NC1=CC=C(Cl)C(C(F)(F)F)=C1)N(CC2)CCN2C3=C4C(SC(C5=CC=C(N6CCN(S(C(C)(C)C)(=O)=O)C(CC(C)C)C6)C=C5)=C4)=NC=N3
N11	O=C(NC1=CC=C(Cl)C(C(F)(F)F)=C1)N(CC2)CCN2C3=C4C(SC(C5=CC=C(N6CCN(S(C(C)(C)C)(=O)=O)C(CCCC(C)C)C6)C=C5)=C4)=NC=N3
N12	O=C(NC1=CC=C(Cl)C(C(F)(F)F)=C1)N(CC2)CCN2C3=C4C(SC(C5=CC=C(N6CCN(S(C(C)(C)C)(=O)=O)C(CO)C6)C=C5)=C4)=NC=N3
N13	O=C(NC1=CC=C(Cl)C(C(F)(F)F)=C1)N(CC2)CCN2C3=C4C(SC(C5=CC=C(N6CCN(S(C(C)(C)C)(=O)=O)C(CCO)C6)C=C5)=C4)=NC=N3
N14	O=C(NC1=CC=C(Cl)C(C(F)(F)F)=C1)N(CC2)CCN2C3=C4C(SC(C5=CC=C(N6CCN(S(C(C)(C)C)(=O)=O)C(CC(CO)C)C6)C=C5)=C4)=NC=N3
